# A Comparison of In Vivo Bone Tissue Generation Using Calcium Phosphate Bone Substitutes in a Novel 3D Printed Four-Chamber Periosteal Bioreactor

**DOI:** 10.3390/bioengineering10101233

**Published:** 2023-10-21

**Authors:** D. S. Abdullah Al Maruf, Kai Cheng, Hai Xin, Veronica K. Y. Cheung, Matthew Foley, Innes K. Wise, Will Lewin, Catriona Froggatt, James Wykes, Krishnan Parthasarathi, David Leinkram, Dale Howes, Natalka Suchowerska, David R. McKenzie, Ruta Gupta, Jeremy M. Crook, Jonathan R. Clark

**Affiliations:** 1 Integrated Prosthetics and Reconstruction, Department of Head and Neck Surgery, Chris O’Brien Lifehouse, Camperdown, NSW 2050, Australia; maruf.almaruf@lh.org.au (D.S.A.A.M.); hai.xin@lh.org.au (H.X.); cate.froggatt@lh.org.au (C.F.); james.wykes@lh.org.au (J.W.); krishnanpartha@hotmail.com (K.P.); dl@esoms.com.au (D.L.); dale.howes@lh.org.au (D.H.); 2 Central Clinical School, Faculty of Medicine and Health, The University of Sydney, Camperdown, NSW 2050, Australia; 3 Royal Prince Alfred Institute of Academic Surgery, Sydney Local Health District, Camperdown, NSW 2050, Australia; kai.cheng@health.nsw.gov.au; 4 Department of Tissue Pathology and Diagnostic Oncology, NSW Health Pathology, Royal Prince Alfred Hospital, Camperdown, NSW 2050, Australia; veronica.cheung@health.nsw.gov.au (V.K.Y.C.); ruta.gupta@health.nsw.gov.au (R.G.); 5 Sydney Medical School, Faculty of Medicine and Health Sciences, The University of Sydney, Camperdown, NSW 2006, Australia; 6 Sydney Microscopy & Microanalysis, The University of Sydney, Camperdown, NSW 2006, Australia; matthew.foley@sydney.edu.au; 7 Laboratory Animal Services, The University of Sydney, Camperdown, NSW 2050, Australia; innes.wise@sydney.edu.au; 8 Arto Hardy Family Biomedical Innovation Hub, Chris O’Brien Lifehouse, Camperdown, NSW 2050, Australia; will.lewin@lh.org.au (W.L.); david.mckenzie@sydney.edu.au (D.R.M.); jeremy.crook@lh.org.au (J.M.C.); 9 Sarcoma and Surgical Research Centre, Chris O’Brien Lifehouse, Camperdown, NSW 2050, Australia; 10 School of Physics, Faculty of Science, The University of Sydney, Camperdown, NSW 2050, Australia; natalka.suchowerska@sydney.edu.au; 11 Intelligent Polymer Research Institute, AIIM Facility, The University of Wollongong, Wollongong, NSW 2522, Australia; 12 School of Medical Sciences, Faculty of Medicine and Health, The University of Sydney, Camperdown, NSW 2050, Australia

**Keywords:** bioreactor, polyether ether ketone, bone substitute, osteogenesis, 3D printed, vascularized periosteum

## Abstract

Autologous bone replacement remains the preferred treatment for segmental defects of the mandible; however, it cannot replicate complex facial geometry and causes donor site morbidity. Bone tissue engineering has the potential to overcome these limitations. Various commercially available calcium phosphate-based bone substitutes (Novabone^®^, BioOss^®^, and Zengro^®^) are commonly used in dentistry for small bone defects around teeth and implants. However, their role in ectopic bone formation, which can later be applied as vascularized graft in a bone defect, is yet to be explored. Here, we compare the above-mentioned bone substitutes with autologous bone with the aim of selecting one for future studies of segmental mandibular repair. Six female sheep, aged 7–8 years, were implanted with 40 mm long four-chambered polyether ether ketone (PEEK) bioreactors prepared using additive manufacturing followed by plasma immersion ion implantation (PIII) to improve hydrophilicity and bioactivity. Each bioreactor was wrapped with vascularized scapular periosteum and the chambers were filled with autologous bone graft, Novabone^®^, BioOss^®^, and Zengro^®^, respectively. The bioreactors were implanted within a subscapular muscle pocket for either 8 weeks (two sheep), 10 weeks (two sheep), or 12 weeks (two sheep), after which they were removed and assessed by microCT and routine histology. Moderate bone formation was observed in autologous bone grafts, while low bone formation was observed in the BioOss^®^ and Zengro^®^ chambers. No bone formation was observed in the Novabone^®^ chambers. Although the BioOss^®^ and Zengro^®^ chambers contained relatively small amounts of bone, endochondral ossification and retained hydroxyapatite suggest their potential in new bone formation in an ectopic site if a consistent supply of progenitor cells and/or growth factors can be ensured over a longer duration.

## 1. Introduction

A range of pathologies may result in segmental defects of the mandible. Such defects reduce the functionality of the jawbone and may affect facial aesthetics, causing psychosocial problems [1,2,3]. The reconstruction of segmental mandibular defects continues to be an ongoing challenge. Although surgical correction using autologous bone is considered the gold standard, it is difficult to replicate complex facial geometry using autologous bone and its harvest is associated with substantial donor site morbidity [1]. Bone tissue engineering (BTE), using a combination of cells and biomaterials with the desired biochemical and physicochemical properties, is a potential alternative method for repairing these and other critical-sized bone defects [4].

Various metals, permanent and resorbable polymers, ceramics, growth factors, xenografts, and allogeneic bone have been used as substitutes for autologous bone over the last few decades [1,5,6,7,8]. Polyether ether ketone (PEEK) is a fracture-tough and non-resorbable polymer with an elastic modulus that is compatible with bone. It, therefore, is less likely to result in stress-shielding than titanium or ceramics. However, PEEK is inert and does not osseointegrate with neighboring bone when implanted. Several in vitro and in vivo studies have shown that surface treatment of PEEK using plasma immersion ion implantation (PIII) supports cellular attachment, proliferation, osseointegration, osteoconduction, mineralization, and bone formation [9,10,11].

Calcium phosphate is a critical structural component of human bone stored as both calcium hydroxyapatite and beta tricalcium phosphate. Xenogeneic hydroxyapatite from animal bones and phytogenic bone-analog calcium phosphate derived from sea algae have also been used as bone substitute materials [12,13]. In this study, we compared autologous bone with three different commercially available calcium phosphate-based bone substitutes placed in PIII–PEEK bioreactors that were 3D printed, wrapped with vascularized periosteum, and maintained in vivo for 8, 10, or 12 weeks. The bone substitutes tested are used clinically (especially in dentistry for repairing small bone defects around teeth) and include Novabone^®^, a synthetic calcium phosphosilicate, BioOss^®^, a bovine-origin hydroxyapatite, and Zengro^®^, consisting predominantly of calcium phosphate with a carbonate apatite. BioOss^®^, a deproteinized bovine derived bone hydroxyapatite, is an osteoconductive xenograft [14] that has demonstrated good clinical outcomes in craniomaxillofacial applications [15]. It produces the desired synostosis with host bone [16,17]. As BioOss^®^ has poor osteoinduction [18], growth factors can be incorporated with BioOss^®^ to enhance its osteoinductive properties [19]. Our study employed PRF and scapular periosteum, rich in osteogenic growth factors and stem cells, for this purpose. PRF is enriched in bioactive growth factors, including bone morphogenetic protein-2 (BMP-2) [20]. In contrast to BioOss^®^, the active ingredient of Zengro^®^, synthetic carbonate-containing hydroxyapatite (CHA), is a promising ceramic that mimics the chemical composition of native bone tissue [21]. Similar to BioOss^®^, Zengro^®^ was also mixed with PRF to allow for enhanced bioactivity within the bioreactor chamber.

Periosteum is a thin membrane lining the surface of bone. It has a cell-rich inner (cambium) layer and an outer fibrous layer. Periosteum plays an important role in oxygen and nutrient delivery to bone via its vascular network and its cambium layer contains osteoblasts and osteoprogenitor cells, which are essential for bone development and fracture healing [22]. Vascularized periosteal flaps have been effective in inducing bone regeneration [23,24] and when combined with bioreactors made of polymethylmethacrylate filled with autologous bone grafts, the constructs have formed customized bone suitable for reconstructing small ovine mandibular defects using free tissue transfer [25]. In the present work, we describe a novel ovine periosteal flap that is large enough to wrap a customized bone scaffold that could be used to reconstruct large (4 cm) segmental mandibular defects. The study compared osteogenesis and mineral volume in a four chambered in vivo PIII-treated 3D printed PEEK bioreactor with the intention of determining the preferred synthetic bone substitute that could be used in future studies of segmental mandibular repair employing customized scaffolds.

Plasma immersion ion implantation treated PEEK or PIII-treated PEEK has excellent credentials as an osseointegrating material, showing an ability to encourage the adherence and mineralization of bone cells in vitro [11] as well as osseointegration of bone in vivo [9,10]. The PIII-treated PEEK material was chosen to form the bioreactor walls in the present study to ensure there was no inhibition to the migration of bone progenitor and associated cells into the experimental zone and to make sure that conditions were equally conducive to bone formation for each of the materials tested.

## 2. Materials and Methods

### 2.1. Design of the Four-Chambered Bioreactors

The bioreactor model was created using Autodesk 3dsMax 2020 (Autodesk, San Rafael, CA, USA) applying a polygonal modelling technique. The overall volume of the bioreactor was 10 mm × 10 mm × 40 mm. A standard primitive, such as a box of 10 mm × 10 mm × 1 mm, was created as a sidewall of the bioreactor. Two intersected boxes of 13 mm × 40 mm × 1 mm were created and connected to the side box to form a four-chambered bioreactor. Hemisphere and cylinder models were added to each chamber’s internal surface and sidewalls for identification purposes. Chamfer and sub-division functions in Autodesk 3dsMax2020 were utilized to smooth the sharp edges of the bioreactor.

### 2.2. Additive Manufacturing of PEEK Bioreactors

**Figure 1:**
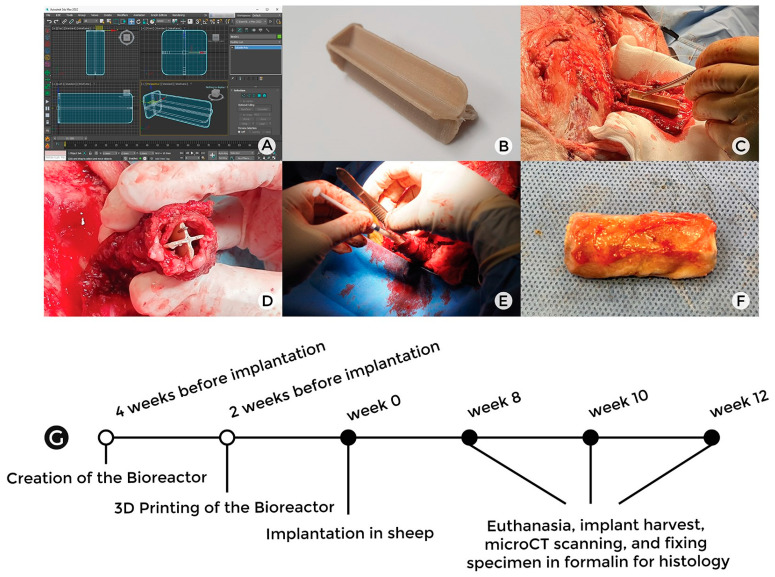
Outline of the experimental protocol. The designing of a four-chambered bioreactor (**A**), followed by 3D printing using PEEK (**B**). The wrapping of the bioreactor with scapular periosteum (**C**,**D**). The filling of the bioreactor chamber with three different bone substitutes and autologous bone, followed by implantation (**E**). Post-euthanasia harvesting of bioreactor sample (**F**). Timeline of the entire study (**G**).

The PEEK Bioreactor Implants (Figure 1A) were sliced for printing with Simplify3D (V4.1.2) and fabricated using an AON-M.2 3D fused deposition modelling (FDM) printer (firmware v3.3.5) from AON3D, Montreal, QC, Canada using 1.75 mm Thermax PEEK (batch 49-080620-06JV) filament produced by 3DXTech, Grand Rapids, MI, USA. A 0.4 mm diameter E3D-V6 Nozzle X and a high-temperature PEI build plate was used for adherence to the platform. The filament was dried for 8 h at 120 °C prior to printing, Z calibration was performed prior to each print, and the constructs were oriented with the base of the design in contact with the raft to eliminate the need for support material. The key printing parameters are listed below in Table 1.

### 2.3. Plasma Immersion Ion Implantation Treatment of PEEK Bioreactors

The PEEK bioreactors were immersed in a dielectric barrier discharge in nitrogen gas at 350 mTorr pressure. The discharge was excited by a high-voltage electrode consisting of a metal electrode covering the bottom and sides of a conical, borosilicate Erlenmeyer flask (250 mL, neck diameter 25 mm). Negative voltage pulses of 10 kV were applied to the electrode using a RUP6 power supply (GBS-Elektronik GmbH, Radeberg, Germany). The pulse frequency was 1500 Hz, the pulse length was 40 μs and the total treatment time was 20 min. After PIII treatment, the PEEK bioreactors were submerged in phosphate-buffered saline (PBS; Gibco/Life Technologies, Macquarie Park, NSW, Australia) overnight. The bioreactors were then washed three times in excess PBS, followed by two washes with excess deionized water, and air-dried overnight at 4 °C. All bioreactors were sterilized in a steam sterilizer before implanting in sheep.

### 2.4. Implantation Surgery

Six female sheep aged 7–8 years and weighing 70–80 kg were distributed in three groups (namely Batch 1, Batch 2, and Batch 3; *n* = 2 sheep per group). Sheep were group housed on straw bedding and fed a standard chaff and hay diet for a minimum of two weeks prior to surgery. All animals were deemed healthy on physical exam prior to surgery. All the procedures were performed with approval from the animal ethics committee (ethics approval number: 2020/1817) of the University of Sydney. Sheep were premedicated with 0.2–0.5 mg/kg methadone (Methodyne^®^, Jurox, Rutherford, NSW, Australia) and 0.2–0.5 mg/kg diazepam (Ilium diazepam, Troy Animal Healthcare, Glendenning, NSW, Australia) via a pre-placed intravenous (IV) canula. After general anesthesia was induced with IV propofol (Propofol-Lipuro^®^ 1%, B. Braun Melsungen AG, Melsungen,, Germany; 2–4 mg/kg) administration, the area over the left scapular infraspinous muscle was shaved, and the surgical site was sterilized with chlorhexidine and povidone iodine. A 20 cm long skin incision was created over the infraspinatus and teres major muscles, which were detached from the scapula preserving a 1 mm cuff of muscle on the periosteum. The periosteal branch of the circumflex scapular artery and vein supplying the lateral border of the scapula were Identified and isolated. Afterwards, the periosteum at tip of the scapula was incised, followed by elevation of the periosteum off the scapula towards the vascular pedicle using a periosteal elevator, taking care not to damage the periosteum or vascular supply. The periosteum was elevated from the entire external surface of the scapula (Figure 1B).

The bioreactors were wrapped with the raised periosteum leaving one end open (Figure 1C). The edges of the periosteum around each implant were sutured with a 4-0 non-resorbable polypropylene suture (Prolene^®^, Johnson & Johnson, North Ryde, NSW, Australia) whilst taking care not to traumatize the vascular pedicle. Next, 40 mL of blood were collected from the sheep during the procedure to prepare platelet-rich fibrin (PRF). Autologous bone grafts were harvested from the scapula, creating a 12 mm diameter full-thickness defect (for a different experiment). Then 1 mL of each bone substitute (Novabone^®^ Osteogenics Biomedical, Lubbock, TX 79424, USA), BioOss^®^ (Geistlich Pharma; Chatswood, NSW, Australia and Zengro^®^ Southern Implants, Irene, RSA, South Africa) and morselized autologous bone graft were mixed with PRF and placed in separate chambers of the bioreactors. The open end of the bioreactor was sealed by suturing the free edge of the periosteum together. Holes were drilled into the spine and scapular tip to reattach the infraspinatus and teres major muscles to the scapula using a 1/0 synthetic absorbable suture composed of 90% glycolide and 10% L-lactide (Vicryl^®^, Johnson & Johnson, North Ryde, NSW, Australia). The bioreactors were secured to overlying muscle with 3-0 Vicryl^®^ to prevent twisting of the pedicle. Subcutaneous fascia and muscle layers were closed with interrupted 3-0 Vicryl^®^ suture and the skin was closed with running subcuticular undyed 3-0 monofilament Poliglecaprone 25 suture (Monocryl^®^, Johnson & Johnson, North Ryde, NSW, Australia) (Figure 1D).

Sheep were monitored for the duration of the study. One sheep from Batch 2 aspirated during the surgical procedure and was euthanized on the same day of implantation. Sheep from Batch 1, Batch 2, and Batch 3 were euthanized at the end of 8, 10, and 12 weeks after the surgery, respectively. Bioreactors were harvested at each timepoint, and the external aspects of the chambers were inked blue, black, yellow, and red to correspond with chambers (1) autologous bone graft, (2) Novabone^®^, (3) BioOss^®^, and (4) Zengro^®^, respectively to allow for orientation under the microscope. Samples were then stored in 10% neutral buffered formalin (NBF) for subsequent fixation of the tissues.

### 2.5. MicroCT Scanning and Analysis of the Harvested PEEK Bioreactor Contents

**Figure 2:**
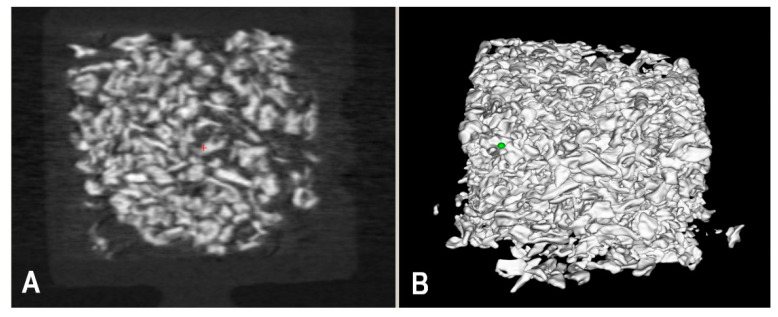
Optimization of microCT scanning method using BioOss^®^. 2D (**A**) and 3D (**B**) representation of microCT scanned images of BioOss^®^.

The harvested bioreactors were removed from the NBF and placed on absorbent paper to remove any excess fluid, and wrapped in parafilm to prevent dehydration during the microCT scanning. The harvested samples were imaged using a Bruker SkyScan 2214 scanner (Bruker MicroCT, Kontich, Belgium) at a resolution of 30 µm, with the source running at 60 kVp and 200 µA. A thin aluminum filter (0.25 mm) was used to minimize the need for beam-hardening correction during reconstruction. One of the chamber contents, BioOss^®^, was microCT scanned within a single chambered bioreactor to optimize the scanning method (Figure 2). The projection images for each scan were reconstructed with identical parameters using NRecon (version 2.1.0.1, Bruker MicroCT). To calculate the mineral density (MD) of the samples during analysis, two hydroxyapatite calibration phantoms (0.250 and 0.750 g/cm^3^) were scanned and reconstructed using the same parameters.

CTAn 1.11.6.0 (Bruker-MicroCT, Kontich, Belgium) software package was used to analyze the volume and density in each bioreactor chamber. Once the datasets of cross-section slices were uploaded in CTAn software, the top (showing the start of mineralized particle/s) and bottom (showing the end of mineralized particle/s) slices to be analyzed were selected using the **Selection Reference** option as the region of interest (ROI) for the analysis. Once the ROI was selected, according to the manufacturer’s protocol, the upper threshold set to 255 and the lower threshold was set at 20 to correspond to the mineralized contents in each chamber on 8-bit (0~255 grey level) bitmap (BMP) images. The analyzing parameters examined were: (1) total tissue volume (TV), (2) mineral volume (MV), and (3) mineral volume fraction (MV/TV). Contents from each segmented chamber were assessed within the selected ROI using an automated 3D analysis plugin provided by the manufacturer. To calibrate MD with Hounsfield units (HU), the reconstructed phantom datasets were included in the calculation based on the assumption that HU_air_ = −1000 and HU_water_ = 0.

### 2.6. Histology

All histology assessments were performed by two Fellows of the Royal College of pathologists of Australasia (FRCPA) in clinical grade National Association of Testing Authorities (NATA) accredited pathology department adhering to National Pathology Accreditation Advisory Council standards (ISO 15189). the bioreactors were sliced into 4 mm thick sections perpendicular to the long-axis of the bioreactor and embedded sequentially from one end to the other into tissue cassettes for an overnight processing schedule on the Leica Peloris Tissue Processor. The sections were embedded into paraffin on the TES Valida^®^ Modular Paraffin Embedding Center, sectioned at a 4 µm thickness with Leica RM2235 Rotary Microtome, and mounted on glass slides for staining with Sakura Tissue-Tek Prisma automated hematoxylin and eosin (H&E) staining machine. The multiple spicules of BioOss^®^ and Zengro^®^ resembling the crush artefact made use of morphometry techniques difficult. Osteogenesis was qualitatively and semi-qualitatively assessed using brightfield microscopy techniques at 1.25X, 4X, 10X, 20X, and 40X magnification by clinical surgical pathologists based on the histologic presence or absence of osteoid, chondroid matrix, granulation tissue, and foreign body reaction. Bone, cartilage, and granulation were identified as per the histologic criteria specified in Wheater’s Functional Histology: A Text and Colour Atlas (6th edition) [26], and Histology for Pathologists (5th edition) [27]. Additionally, Alcian blue (pH 1) staining was also performed on the tissue sections to confirm the cartilage detection as evidenced in the H&E stained images.

### 2.7. Statistical Analysis

Statistical analyses were performed with GraphPad Prism 9 software (GraphPad Software Inc, San Diego, CA, USA). Data generated by microCT image analysis were compared using two-way analysis of variance (ANOVA) with Tukey multiple comparisons test to determine the effect at various timepoints (each timepoint (8 weeks, 10 weeks, and 12 weeks) has two sheep per group with eight chambers in total; however, the 10 week timepoint has one sheep per group with four chambers in total). Random effects modelling (robust to missing data) was used to compare the mineral volume (MV) between chambers at all timepoints adjusting for time and correlated data. *p* < 0.05 was considered statistically significant.

## 3. Results

### 3.1. Histology

**Figure 3:**
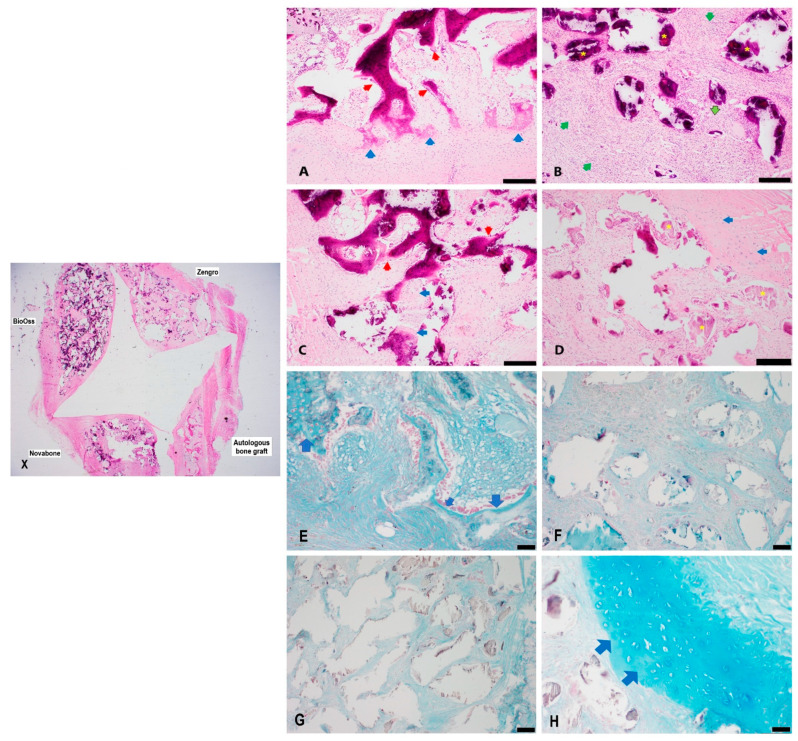
Histological evaluation of newly formed tissue in different groups at 8 weeks post-implantation. Low magnification view of different bone substitutes in the bioreactor chambers (**X**). Histological evaluation of autologous bone graft (**A**), Novabone^®^ (**B**), BioOss^®^ (**C**), and Zengro^®^ (**D**) at 8 weeks post-implantation. The autologous bone graft showed the most amount of bone formation that appeared to be arising from endochondral ossification. Novabone^®^ showed a predominantly foreign body reaction with a chronic inflammatory infiltrate of histiocytes and lymphocytes in the stroma around the implanted material. Focal/mild bone formation and early cartilage (chondroid) areas were seen in BioOss^®^ and Zengro^®^ (red arrow—bone; blue arrow—chondroid/cartilage; green arrow—inflammatory histiocytic and lymphocytic infiltrate; outlined green arrow—foreign body multinucleated giant cells; yellow star—implanted material). Scale bar: 200 µm. A higher magnification view of neo-cartilage formed within the autologous bone graft group (**E**) and Zengro^®^ (**H**) stained with Alcian blue (pH:1) (Blue arrow—cartilage). No obvious neo-cartilage tissue was observed in Novabone^®^ (**F**). Neo-cartilage in BioOss^®^ (**G**) group was too focal/too small and cut out on deeper levels, hence, no cartilage tissue was identified on Alcian blue-stained tissue sections. Scale bar: 100 µm.

**Figure 4:**
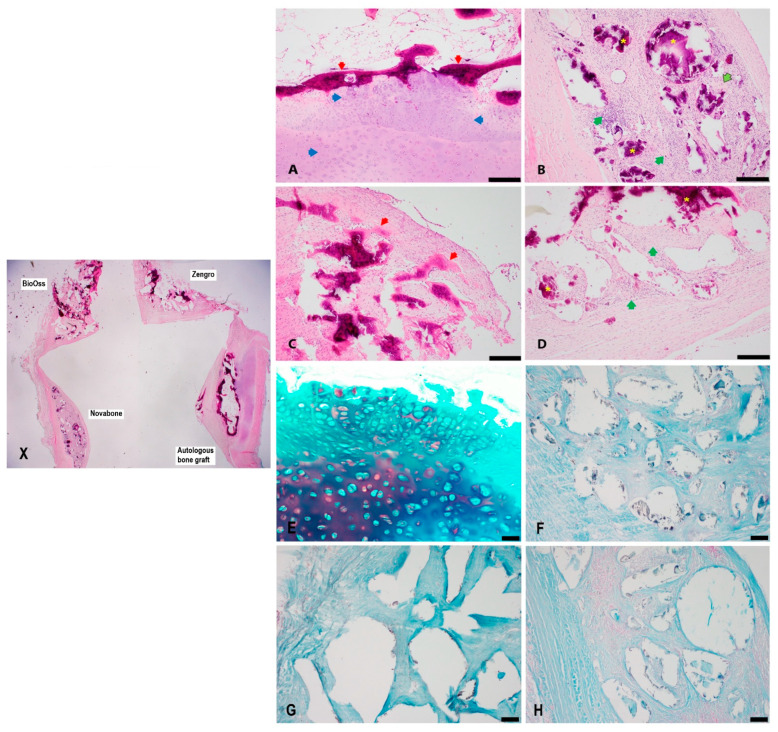
Histological evaluation of newly formed tissue in different groups at 10 weeks post-implantation. Low magnification view of different bone substitutes in the bioreactor chambers (**X**). Histological evaluation of autologous bone graft (**A**), Novabone^®^ (**B**), BioOss^®^ (**C**), and Zengro^®^ (**D**) at 10 weeks post-implantation, see Supplementary Materials. The autologous chamber showed endochondral ossification with a broad area of cartilaginous tissue leading to a layer of bone formation (**A**). Both the Novabone^®^ (**B**) and Zengro^®^ (**D**) chambers only showed inflammatory and foreign body reactions to the implanted material. Focal bone formation was seen in the BioOss^®^ chamber (**C**) (red arrow—bone; blue arrow—chondroid/cartilage; green arrow—inflammatory histiocytic and lymphocytic infiltrate; outlined green arrow—foreign body multinucleated giant cells; yellow star—implanted material). Scale bar: 200 µm. Higher magnification view of neo-cartilage formed within the autologous bone graft group (**E**), stained with Alcian blue (pH:1). No obvious neo-cartilage tissue was identified in the Alcian blue stained tissue sections from Novabone^®^ (**F**), BioOss^®^ (**G**), and Zengro^®^ (**H**) group at 10 weeks post-implantation. Scale bar: 100 µm.

**Figure 5:**
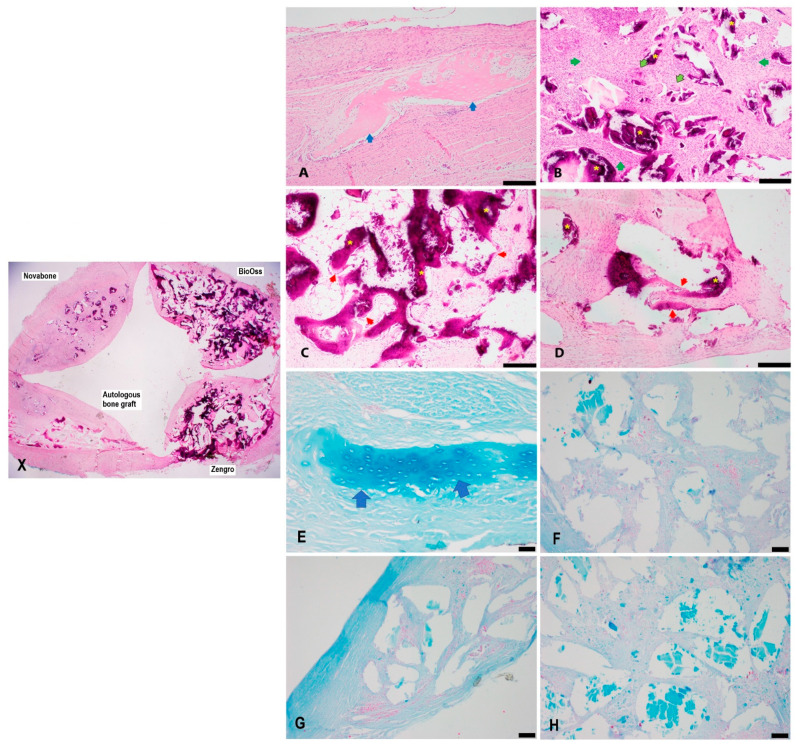
Histological evaluation of newly formed tissue in different groups at 12 weeks post-implantation. Low magnification view of different bone substitutes in the bioreactor chambers (**X**). Histological evaluation of autologous bone graft (**A**), Novabone^®^ (**B**), BioOss^®^ (**C**), and Zengro^®^ (**D**) at 12 weeks post-implantation. The autologous chamber showed focal bone and cartilage (pictured) formation (**A**). Novabone^®^ material elicited an inflammatory foreign body reaction only within the chamber (**B**). Both BioOss^®^ (**C**) and Zengro^®^ showed focal bone formation, which can be seen as a thin layer of osteoid surrounding the edges of the implanted material (**D**) (red arrow—bone; blue arrow—chondroid/cartilage; green arrow—inflammatory histiocytic and lymphocytic infiltrate; outlined green arrow—foreign body multinucleated giant cells; yellow star—implanted material). Scale bar: 200 µm. Higher magnification view of neo-cartilage formed within the autologous bone graft group (**E**), stained with Alcian blue (pH:1) (blue arrow—cartilage). No obvious formation of neo-cartilaginous tissue was observed in the Novabone^®^ (**F**), BioOss^®^ (**G**), and Zengro^®^ (**H**) groups. Scale bar: 100 µm.

H&E-stained bioreactor chambers were semi-quantitatively evaluated microscopically at 8-, 10-, and 12-week timepoints as summarized in Table 2. All the chambers showed varying degrees of histiocytic and foreign body reactions to the implanted material (Figure 3, Figure 4 and Figure 5). Histological examination demonstrated the most consistent bone formation within the autologous graft chamber (Figure 3A). Ossification was predominantly endochondral for the autologous, BioOss^®^, and Zengro^®^ chambers (Figure 3A,C–E,H, Figure 4A,E, and Figure 5A,E. The BioOss^®^ and Zengro^®^ chambers showed less bone formation compared to autologous bone with no specific relation to the duration of implantation (5C, and 5D). Cartilage formation in both BioOss^®^ and Zengro^®^ groups was observed only at earlier timepoint (8 weeks post-surgery) (Figure 3C,D,H). However, the cartilaginous tissue formed in the BioOss^®^ group was too focal/too small and cut out on deeper levels, so there was no cartilage tissue remaining to see on Alcian blue staining. Novabone^®^ did not show any morphologic evidence of bone or cartilage formation.

### 3.2. microCT Analysis of the Bioreactor Contents

**Figure 6:**
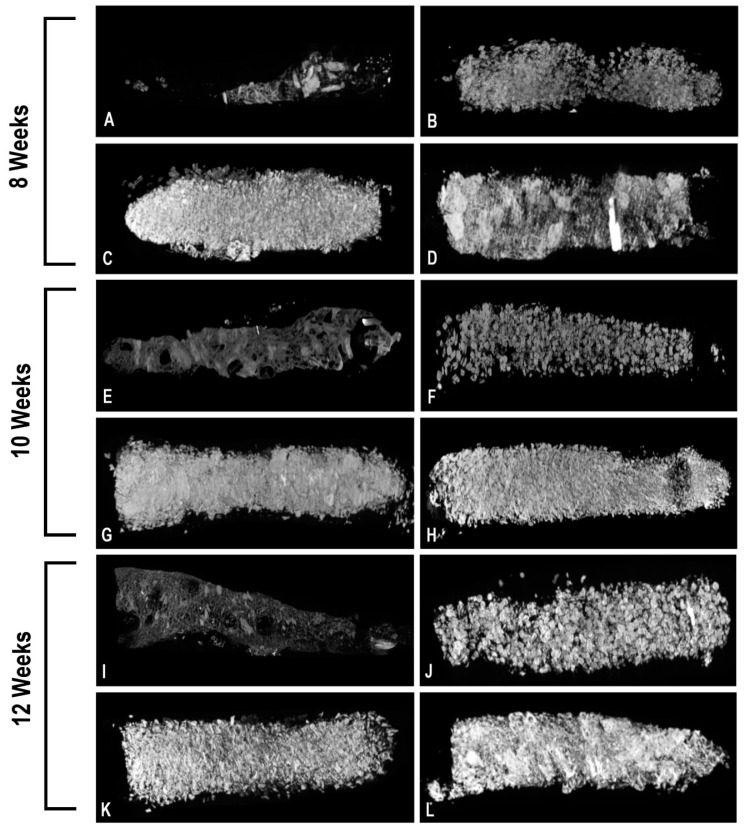
3D reconstructed microCT images of bone substitutes filled in each bioreactor chamber at various timepoints. Autologous bone graft in chamber 1 (**A**,**E**,**I**), Novabone^®^ in chamber 2 (**B**,**F**,**J**), BioOss^®^ in chamber 3 (**C**,**G**,**K**), and Zengro^®^ in chamber 4 (**D**,**H**,**L**).

**Figure 7:**
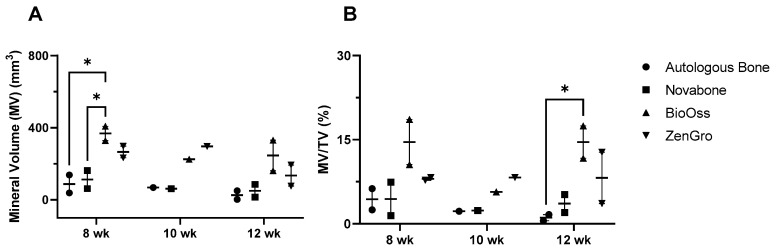
Analysis of the mineral volume (**A**) and mineral volume–total tissue volume ratio (**B**) measured by microCT in the bioreactor chambers at 8-, 10-, and 12-week timepoints. Results show mean ± standard error of the mean. * Indicates *p* < 0.05.

Data for mineral volume (MV) and mineral volume/total tissue volume ratio (MV/TV) and mineral density (MD) were generated by analyzing the 3D reconstruction of each chamber’s contents at 8 weeks, 10 weeks, and 12 weeks post-surgery as shown in Figure 6. A two-way ANOVA revealed that the mean MV in the BioOss^®^ experimental group (368.4 mm^3^) was significantly higher than the autologous bone group (88.1 mm^3^) at the 8-week timepoint (*p* = 0.017) as shown in Figure 7A. However, there was no significant difference in mineral volume between the Novabone^®^ (112.5 mm^3^) and Zengro^®^ groups (265.6 mm^3^) compared to autologous bone at the 8-week timepoint. There was no significant difference in mineral volume at the 10- and 12-week timepoints among the groups (Figure 7A). Furthermore, although no significant difference was observed in mineral volume–total tissue volume ratio among the groups at earlier timepoints, the ratio in BioOss^®^ was significantly higher (14.6%) compared to that of the autologous bone group (1.1%) at the 12-week timepoint (*p* = 0.035) (Figure 7B).

**Figure 8:**
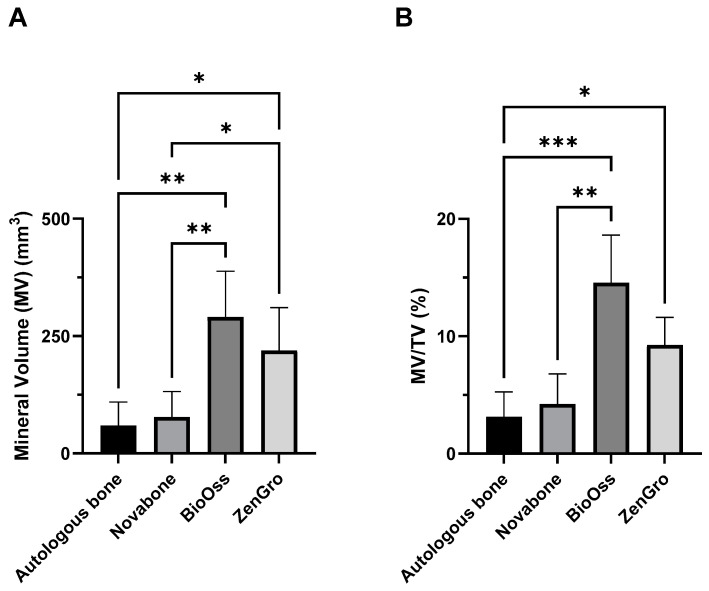
Analysis of mineral volume (**A**) and mineral volume–total tissue volume ratio (**B**) for various groups by combining measures of all timepoints. Results show mean ± standard error of the mean. * Indicates *p* < 0.05, ** indicates *p* < 0.01, and *** indicates *p* < 0.001.

Adjusted analyses of the combined data demonstrated that both BioOss^®^ and Zengro^®^ were associated with higher mineral volume or MV (BioOss^®^: 290.9 mm^3^ and Zengro^®^: 219.4 mm^3^) compared to autologous bone graft (MV: 59.6 mm^3^), *p* = 0.001 and 0.02, respectively. BioOss^®^ and Zengro^®^ also demonstrated higher mineral volume–total tissue volume ratio or MV/TV (BioOss^®^: 14.6% and Zengro^®^: 9.3%) compared to autologous bone graft (MV/TV: 3.2%), *p* < 0.001 and 0.046, respectively. BioOss^®^ and Zengro^®^ also demonstrated higher MV compared to Novabone^®^ (MV: 77.6 mm^3^), *p* = 0.002 and 0.042, respectively, and BioOss^®^ demonstrated higher MV/TV compared to Novabone (MV/TV: 4.2%), *p* = 0.001 (Figure 8A,B). However, no statistically significant change was observed over time in any of the experimental group for any of the analyzed parameters.

**Figure 9:**
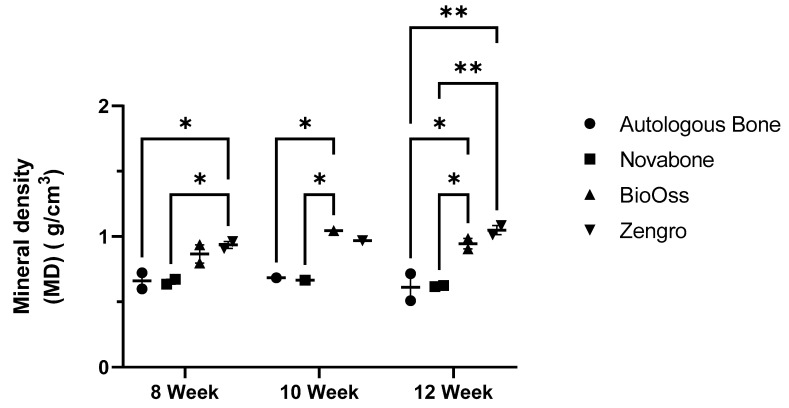
Quantitative analysis of the mineral density in each bioreactor chamber containing various bone substitutes at 8, 10, and 12 weeks post-implantation. Results show mean ± standard error of the mean. * Indicates *p* < 0.05, ** indicates *p* < 0.01.

At 8 weeks, the highest mineral density (MD) was observed in the bioreactor chamber containing Zengro^®^ (0.94 g/cm^3^). This was significantly higher than autologous bone (0.66 g/cm^3^) and Novabone^®^ (0.65 g/cm^3^), *p* = 0.028 and 0.025, respectively (Figure 9). MD in the BioOss^®^ chamber (0.87 g/cm^3^) was not significantly different compared to autologous bone, Novabone^®^, and Zengro^®^ chambers. In contrast, at 10 weeks, the highest MD was observed in the BioOss^®^ chamber (1.05 g/cm^3^). This was significantly higher than autologous bone (0.68 g/cm^3^) and Novabone^®^ (0.67 g/cm^3^), *p* = 0.04 and 0.03, respectively, while the MD in the Zengro^®^ chamber (0.97 g/cm^3^) was not significantly different to other chambers. At 12-weeks post-implantation, compared to autologous bone (0.61 g/cm^3^) and Novabone^®^ (0.62 g/cm^3^), MD was significantly higher in both the BioOss^®^ (0.95 g/cm^3^; *p* = 0.01 and 0.011, respectively) and Zengro^®^ chambers (1.05 g/cm^3^; *p* = 0.002 and 0.002, respectively). However, no statistically significant change was observed over time in any of the experimental groups for mineral density.

## 4. Discussion

Autologous bone is the current gold standard for bone defect repair; however, there are several significant drawbacks to using autologous bone, including donor site morbidity and difficulties replicating complex facial anatomy [25]. The current sheep study compared three commercially available bone substitutes contained in a 3D printed plasma immersion ion implantation treated PEEK bioreactor that was wrapped with vascularized periosteum to determine the best suitable bone substitute to combine with customized scaffolds in future experiments. Specimens from all the groups were evaluated radiologically and histologically. BioOss^®^ and Zengro^®^ chambers, through radiological evaluation, demonstrated higher mineral volume and density compared to autologous bone graft and Novabone^®^, respectively. However, the histological analysis showed that the autologous bone graft chamber had improved bone morphology and that much of the mineralized tissue in the other chambers was not bone, but rather retained graft material. Despite this, there was evidence of osteogenesis and endochondral ossification in the BioOss^®^ and Zengro^®^ chambers. This combined with the higher mineral volume, which may act as a scaffold for future osteogenesis, warrants further exploration of their use as a bone substitute over longer implantation periods.

BioOss^®^, itself, is not an osteoinductive bone substitute; rather, it is an osteoconductive material [28]. Therefore, this study employed the strategy of adding PRF to BioOss^®^, as well as to other the bone substitutes in different chambers of the bioreactor with an aim introduce osteoinduction (except the Novabone^®^, which was used in its original semi-gel-like form). In fact, the addition of PRF to hydroxyapatite and calcium-based scaffolds can enhance bone regeneration by promoting the growth and proliferation of bone cells, leading to faster and more effective repair of the bone defect [29,30]. This was further supported by Piattelli et al. (1999), who observed new bone formation surrounding the BioOss^®^ particles in human maxillary sinus augmentation procedures [31]. In the study of Piattelli, the BioOss^®^ particles functioned as a resorbable scaffold that facilitated the growth of new bone tissue around the graft particles in the augmented area [31]. Similar application of BioOss^®^ as a scaffold have also been reported by Lee YC et al. 2019 [28]. One of the key benefits of ceramic porous scaffolds is their ability to facilitate bone ingrowth. This means that, as the scaffold degrades over time, new bone tissue grows into the pores of the scaffold, eventually replacing it completely. This process can help to repair both load-bearing and non-load-bearing bone defects, depending on the location and severity of the injury [21,32]. Interestingly, BioOss^®^ can also be made osteoinductive by adding osteoinductive components, including various stem cells [28]. Similar findings of new bone formation and level of osteogenesis around the ceramic particles have also been reported with bone substitute containing calcium phosphate having a carbonated apatite [21]. However, this was in a bone defect model and not in an ectopic site, and in fact, this bone substitute in previous studies was not used in a confined chamber to regenerate new bone tissue. Although microCT analysis showed significantly increased mineralized material in the BioOss^®^ and Zengro^®^ chambers, histological results revealed that most of this mineralized tissue was retained calcium/carbonate-containing hydroxyapatite. An important histology finding in this study was the presence of cartilaginous tissue, typical of endochondral ossification, which suggests that the retained hydroxyapatite may facilitate osteogenesis with additional time. The PRF–hydroxyapatite mixture receives continuous growth factor stimulation from the PRF and vascularized periosteum, bringing progenitor cells from the cambium layer of the periosteum that, with the aid of the calcium phosphate, initiate osteogenesis. However, the presence of cartilage in the chambers was inconsistent and independent of the duration of the study. Endochondral ossification naturally occurs during bone fracture healing and may enhance bone regeneration in hypoxic conditions compared to intramembranous ossification [33]. The bioreactor chambers in this study are initially avascular until neovascularization from the periosteum occurs, which has potential progenitor cell sources in the cambium layer [34]; this low-oxygen environment may have contributed to the predominantly endochondral ossification observed in the autologous bone, BioOss^®^, and Zengro^®^ chambers. Despite the autologous bone chamber having superior bone morphology, the Novabone^®^, BioOss^®^, and Zengro^®^ chambers had greater total tissue volume on microCT and histology. This probably represents increased bioavailability of the autologous bone leading to increased mineral resorption.

This study employed scapula bone as autologous bone graft, which is a flat bone type composed of thin outer layers of cortical bone and thicker cancellous bone. Cancellous bone has more cellular components compared to the mineral contents [35,36]. Hence, this may explain the lower mineral density observed in the autologous bone chamber as observed on microCT evaluation. However, the cellular components indeed help in regenerating new bone tissue, which, over time, can become mineralized. In contrast, as BioOss^®^ and Zengro^®^ are granular, the porosity between granules may act as an internal scaffold that fills with proteinaceous serum, which may result in increased volume and density. However, this did not necessarily translate to bone formation, as we could only see focal osteoid being laid down around the aggregates of these materials. Li Y et al. (2022) conducted a meta-analysis to provide a comprehensive evaluation of new bone formation between BioOss^®^ and autologous demineralized dentin matrix, which demonstrated a comparable outcome between them [37]. Furthermore, BioOss^®^ has also been reported to enhance bone regeneration after a lateral ridge augmentation in a clinical study [38,39]. Although previous studies reported the process of making BioOss^®^ osteoinductive, as well as their efficacy in bone formation [28], these studies were conducted to repair a bone defect or in an environment surrounded by native bone, which ensures a consistent supply of growth factors and/or progenitor cells. Our current study employed vascularized periosteum to supply growth factors and progenitor cells, which can stimulate the osteogenic process. However, the osteogenic potential of periosteum is inferior to that of native bone (unpublished results from another study conducted by our research group). Therefore, the autologous bone graft chamber might have superior osteoinductive properties (progenitor cells and growth factors) from the very beginning compared to other bone substitute chambers, enhancing overall osteogenesis at the end of the study, determined by the histological analysis. Although this study has shown promising radiological findings which may translate into osteogenesis given sufficient time, it is important to acknowledge several limitations that should be addressed in future research. One key limitation is the absence of baseline data on the tissue volume, mineral volume, and mineral density of each chamber contents. This study, as described earlier, employed a four-chambered bioreactor containing different bone substitutes, wrapped with vascularized scapular periosteum in a live ovine model. Therefore, it was not possible to perform a baseline microCT scanning to determine the mineral volume and density. Therefore, the bioreactor design should be modified in future studies, which will allow access to the baseline microCT scanning prior to the animal study, which should be included in similar comparative analyses. Additionally, the small sample size in this study represents another significant limitation, necessitating a larger sample size in the next phase of the research to achieve greater statistical power. Future studies should explore the use of BioOss^®^ and Zengro^®^ as bone substitutes over longer periods and consider modifications that may enhance bone regeneration. This includes utilizing smaller particle sizes to improve calcium bioavailability, incorporating bioactive materials such as BMP-2, and integrating pre-differentiated osteogenic stem cells. A significant advantage of the histologic interpretation by clinical surgical pathologists is that it allows identification and robust analysis of unexpected findings such as the development of immature cartilage as observed in this study. However, utilizing quantitative morphometry techniques suitable for material composed of multiple spicules resembling bone to complement the analysis should also be considered. Furthermore, the design of a porous bioreactor could facilitate better contact between the chamber contents and periosteum, enabling the improved diffusion of nutrients and growth factors within the chamber. An intriguing avenue for investigation would be to compare the role of periosteum versus bone in bone regeneration. Understanding the specific contributions of each elements could provide valuable insights into optimizing bone regeneration techniques.

Addressing these limitations and incorporating these proposed modifications in future studies will enhance the overall understanding of bone regeneration and contribute to the advancement of innovative techniques in this field.

## 5. Conclusions

Bone formation mediated by the autologous bone grafts in this study was moderate, whereas bone formation mediated by BioOss^®^ and Zengro^®^ was low at the timepoints used in this study. No bone formation was observed in the Novabone^®^ chambers. Although both BioOss^®^ and Zengro^®^ demonstrated a low level of ectopic bone formation within the BioOss^®^ and Zengro^®^ chambers, the presence of endochondral ossification and retained hydroxyapatite contents in those chambers possibly suggest their potential in new bone formation in an ectopic site if a consistent supply of progenitor cells and/or growth factors can be ensured over a longer duration.

## Figures and Tables

**Table 1:** Printing parameters.

Nozzle Temperature	405 °C
Platform temperature	170 °C
Chamber temperature	120 °C
Layer height	0.25 mm
Extrusion width	0.5 mm
Print speed	30 mm/s
Outline underspeed	70%
Shells	2
Infill percentage	100%
Infill pattern: raster angles	Rectilinear: 45°, −45°
Raft	3 layers of PEEK, 100% density

**Table 2:** Histological findings of bone and/or cartilage formation within the bioreactor chambers at different timepoints.

Week	Histological Features	Autologous Bone	Novabone^®^	BioOss^®^	Zengro^®^
8.	Bone	+++	−	−	−
	Cartilage	+	−	−	+
	Foreign body reaction	+	++	+	+
8	Bone	+++	−	+	+
	Cartilage	−	−	+	−
	Foreign body reaction	+	++	+	+
10	Bone	++	−	+	−
	Cartilage	++	−	−	−
	Foreign body reaction	+	++	+	+
12	Bone	−	−	−	−
	Cartilage	−	−	−	−
	Foreign body reaction	+	++	+	+
12	Bone	+	−	+	+
	Cartilage	+	−	−	−
	Foreign body reaction	+	++	+	+

Formation and reactions were graded as (+) mild, (++) moderate, (+++) marked, and (−) nil formation.

## Data Availability

The authors do not have permission to share data.

## Supplementary Materials

The following supporting information can be downloaded at: https://www.mdpi.com/article/10.3390/bioengineering10101233/s1. Figure S1: Histological evaluation of neo-cartilage tissue formed within the autologous bone graft (A,C), and Zengro® (B,D) at 10-week post-implantation stained with Alcian Blue technique (pH 1). Scale bar: 200 μm (A,B), and 100 (C,D). Blue colour determines the presence of cartilage tissue with the tissue.

## Abbreviations

3D	Three dimensional
PIII	Plasma immersion ion implantation
PEEK	Polyether ether ketone
BTE	Bone tissue engineering
BMP-2	Bone morphogenetic protein-2
PRF	Platelet-rich fibrin
NBF	Neutral buffered formalin
microCT	micro computed tomography
TV	Total tissue volume
MV	Mineral volume
MV/TV	Mineral volume–total tissue volume ratio
MD	Mineral density
HU	Hounsfield units
H&E	Hematoxylin and eosin
