# Supplementary material for: A Comparison of In Vivo Bone Tissue Generation Using Calcium Phosphate Bone Substitutes in a Novel 3D Printed Four-Chamber Periosteal Bioreactor
Source: Bioengineering (Basel). 2023 Oct 21;10(10):1233. doi: 10.3390/bioengineering10101233 (PMC10604717; doi:10.3390/bioengineering10101233)
Supplement: Supplementary file 1 [file bioengineering-10-01233-s001.zip › bioengineering-2519157-supplementary.pdf]

Supplementary Figure

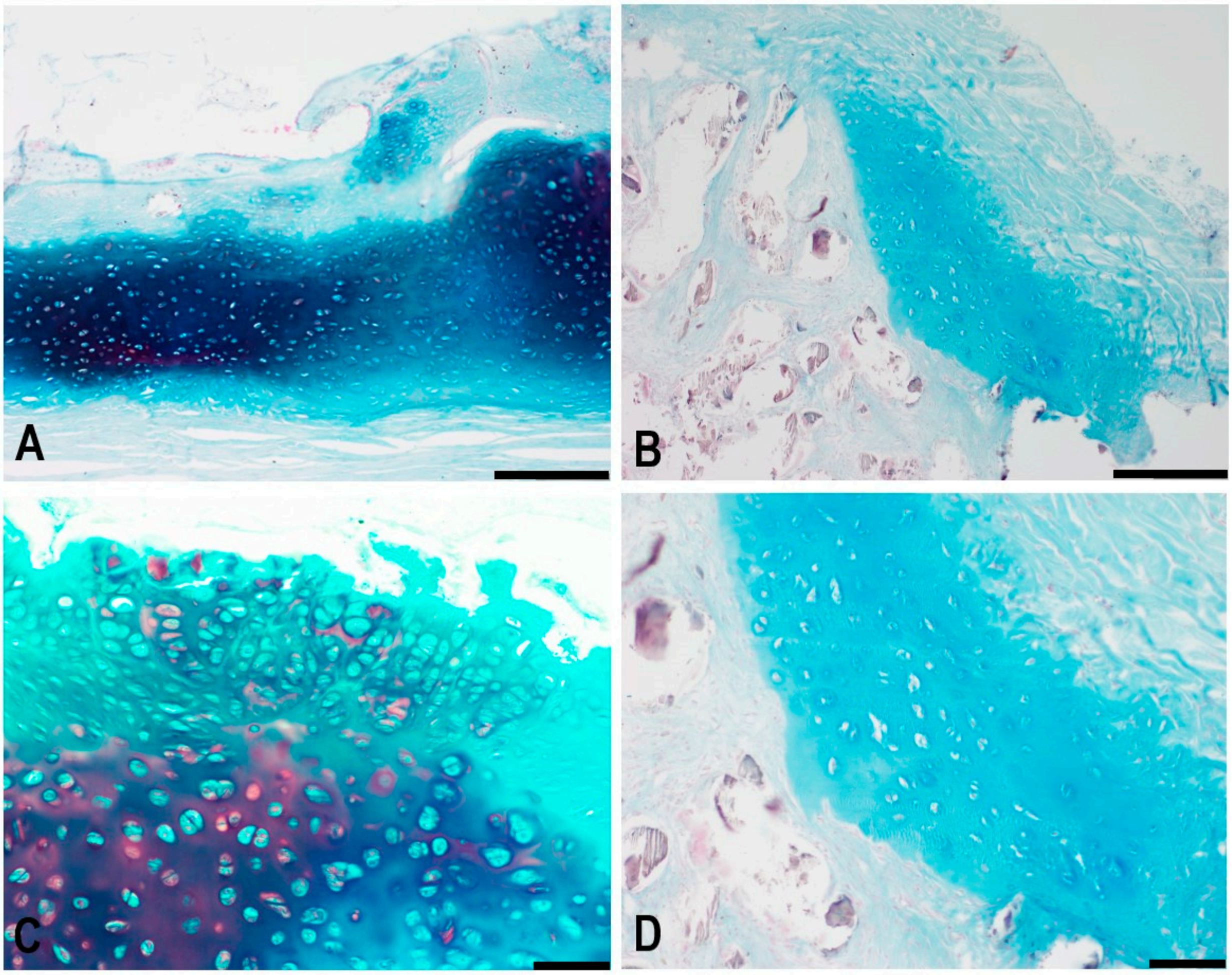

Supplementary Figure S1: Histological evaluation of neo-cartilage tissue formed within the autologous bone graft (A, C), and Zengro® (B, D) at 10-week post-implantation stained with Alcian Blue technique (pH 1). Scale bar: 200 µm (A, B), and 100 (C, D). Blue colour determines the presence of cartilage tissue with the tissue.
